# Malaysian undergraduate psychological well-being and academic motivation dataset: a cross-sectional survey on coping mechanisms and student life stress

**DOI:** 10.3389/fpsyg.2026.1724570

**Published:** 2026-04-17

**Authors:** Siva Sunasundram, Azrul Fazwan Kharuddin, Cheok Mui Yee

**Affiliations:** 1 Faculty of Science & Technology, Spectrum International University College, Selangor, Malaysia; 2 Tun Razak Graduate School, Universiti Tun Abdul Razak, Kuala Lumpur, Malaysia

**Keywords:** academic motivation, coping mechanisms, psychological well-being, student-life stress, undergraduate students

## 1 Introduction

The *Malaysian Undergraduate Psychological Well-Being and Academic Motivation Dataset (MUPWAM)* provides an open-access collection of quantitative data examining the interrelationships among psychological wellbeing, academic motivation, coping mechanisms, and student-life stress among undergraduate students enrolled in higher education institutions in Malaysia. The dataset was developed to facilitate secondary analyses, replication studies, and cross-cultural comparisons, particularly in regions where such data remain limited.

University students globally face increasing mental health challenges driven by academic workload, financial strain, and social adaptation pressures. Recent reviews and meta-analytic evidence highlight rising concerns regarding student wellbeing and motivation in higher education contexts. In Malaysia, however, empirical research examining the intersection of psychological wellbeing and academic motivation remains relatively underdeveloped. This dataset therefore contributes an empirically grounded resource for exploring the dynamics of stress, coping, and motivation among Malaysian undergraduates, serving educational researchers, policymakers, and mental health practitioners.

Consistent with open science principles, the dataset employs standardized and widely validated instruments, including Ryff's Psychological Well-Being Scale (Ryff, 1989), the Academic Motivation Scale (Vallerand et al., 1992), the Student-Life Stress Inventory (Gadzella, 1994), and the Brief COPE Scale (Carver, 1997). These instruments have been widely used across cultural contexts and are appropriate for assessing psychosocial functioning within Malaysian higher education settings.

Importantly, the dataset is based on a cross-sectional design, and therefore supports associational (correlational) analyses only. Any findings derived from this dataset should not be interpreted as causal. Additionally, the use of self-reported measures may introduce common method variance (CMV), which should be considered when conducting advanced statistical analyses.

Beyond its primary purpose, the dataset offers opportunities for subgroup comparisons (e.g., gender or academic discipline), predictive modeling, and psychometric validation within multicultural populations. Researchers are encouraged to apply advanced techniques such as measurement invariance testing and latent variable modeling when reusing the dataset.

All data are anonymized and comply with institutional ethical standards. The dataset is deposited at: https://doi.org/10.5281/zenodo.16964826 and is freely available under a Creative Commons Attribution (CC BY 4.0) license.

## 2 Methodology

### 2.1 Overview

The MUPWAM dataset was compiled using a quantitative cross-sectional research design to capture the psychosocial characteristics of undergraduate students in Malaysia. The dataset includes measures of psychological wellbeing, academic motivation, coping strategies, and student-life stress. While the design is appropriate for descriptive and correlational analyses, it inherently limits causal inference.

All procedures adhered to established ethical and methodological standards for human-subject research, ensuring data integrity, transparency, and reusability.

### 2.2 Data collection period and setting

Data were collected between March and June 2024 from undergraduate students enrolled in public and private universities across Peninsular and East Malaysia. The survey was administered online using Google Forms to maximize accessibility and participation.

Participants were recruited through institutional mailing lists, student organizations, and academic social media platforms. Participation was voluntary, and informed consent was obtained prior to survey completion.

### 2.3 Participants and sampling

A non-probability convenience sampling approach was employed, which is suitable for exploratory research but limits generalizability. As such, the sample may not fully represent the broader population of Malaysian undergraduates.

Inclusion criteria were:

- Enrollment in a full-time undergraduate program
- Age between 18 and 30 years

A total of 368 responses were collected, of which 340 valid cases were retained after data screening. Demographic variables include gender, age, academic year, and faculty cluster (e.g., education, business, engineering, social sciences).

### 2.4 Instruments and measures

The dataset incorporates the following validated psychometric instruments:

- Psychological WellBeing: Measured using Ryff's (1989) 18-item scale assessing autonomy, environmental mastery, personal growth, positive relations, purpose in life, and self-acceptance.
- Academic Motivation: Measured using the Academic Motivation Scale—College Version (Vallerand et al., 1992), capturing intrinsic motivation, extrinsic motivation, and amotivation.
- Coping Mechanisms: Assessed using the Brief COPE (Carver, 1997), which includes adaptive and maladaptive coping strategies.
- Student-Life Stress: Measured using the Student-Life Stress Inventory (Gadzella, 1994), assessing academic, personal, and environmental stressors.

All instruments were selected based on their extensive validation in prior research and relevance to higher education contexts. Where applicable, instruments were linguistically reviewed and adapted, following standard translation and back-translation procedures to ensure clarity and cultural appropriateness for Malaysian respondents.

A pilot test was conducted with a small group of students to ensure item clarity and survey functionality prior to full deployment.

Internal consistency reliability was high, with Cronbach's alpha values ranging from 0.82 to 0.94 across constructs.

### 2.5 Data cleaning and processing

Data were exported into CSV format and processed using IBM SPSS (v27). Cases with more than 10% missing data were removed. Reverse-coded items were corrected, and composite scores were computed for each subscale.

For the remaining dataset, mean substitution was applied to handle minimal missing values. This approach was selected to preserve sample size for descriptive and correlational analyses. However, it may introduce bias, particularly in multivariate analyses. Future users are encouraged to consider more robust methods such as multiple imputation or expectation-maximization algorithms.

No personally identifiable information was collected, ensuring full anonymity.

### 2.6 Data structure and file organization

The dataset is organized into three main files:

- *MUPWAM_Data.csv*: Cleaned dataset with all responses and computed scores
- *MUPWAM_Codebook.pdf* : Detailed variable descriptions and coding schemes
- *MUPWAM_ReadMe.txt*: Metadata, methodology summary, and usage instructions

A summary table mapping constructs, instruments, and item counts has been included to enhance usability.

### 2.7 Reuse and interpretation

The dataset is suitable for:

- Correlational and regression analyses
- Cross-cultural comparisons
- Psychometric validation studies

However, users should note the following:

- Results should be interpreted as associational, not causal
- Common method variance (CMV) may affect findings due to self-report measures
- Sampling limitations restrict generalizability

For advanced analyses, users are encouraged to:

- Test measurement invariance across subgroups
- Apply structural equation modeling (SEM) or latent variable approaches
- Consider statistical remedies for CMV (e.g., marker variables, latent method factors)

### 2.8 Repository information

The dataset is publicly available via Zenodo:

doi: 10.5281/zenodo.16964826

(This link will be updated with a permanent DOI upon publication.)

The dataset is distributed under the Creative Commons Attribution 4.0 International License (CC BY 4.0), permitting unrestricted use with appropriate citation.

## 3 Data description and basic analysis

### 3.1 Dataset overview

The Malaysian Undergraduate Psychological Well-Being and Academic Motivation Dataset (MUPWAM) comprises data from 340 undergraduate students drawn from a range of academic disciplines and higher education institutions across Malaysia. The sample includes respondents from both public and private universities, offering a heterogeneous though not nationally representative representation of the Malaysian tertiary education context due to the use of non-probability convenience sampling.

The dataset captures four primary psychosocial constructs psychological wellbeing, academic motivation, coping mechanisms, and student-life stress each measured using established psychometric instruments with multiple subdimensions. These instruments were selected based on their extensive validation in prior international research and their conceptual relevance to understanding student adjustment within the Malaysian higher education context, where academic pressure, socio-cultural expectations, and transitional stressors are particularly salient.

Variables are systematically coded in numeric and labeled formats to facilitate compatibility with standard statistical software. To further enhance usability, a summary table mapping constructs, original scale references, and item counts has been included in the manuscript. In addition, a conceptual schema illustrating dataset structure (variables, subscales, and file organization) is provided, where permitted by the journal, to improve clarity and facilitate reuse.

Demographically, approximately 60% of participants were female and 40% male, with a mean age of 21.4 years (SD = 2.1). The distribution across academic years was as follows: first-year (28%), second-year (33%), third-year (26%), and fourth-year or above (13%). Academic disciplines represented include social sciences (34%), business and management (27%), education (21%), and engineering/science (18%).

**Figure 1:**
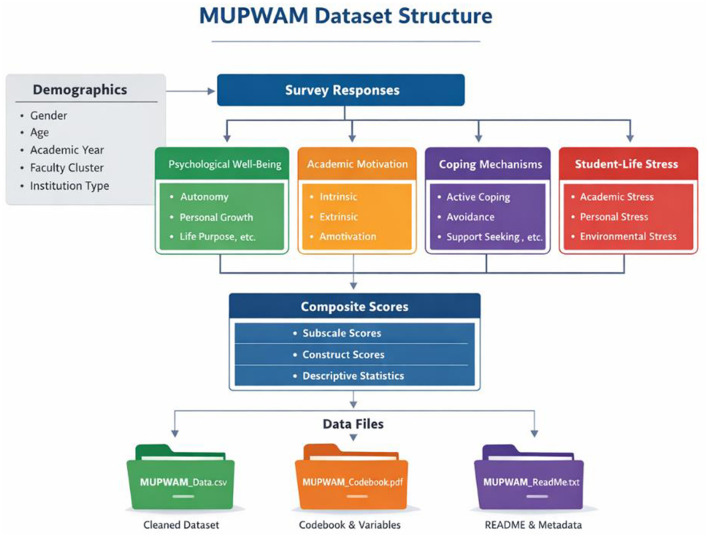
Conceptual structure of the Malaysian Undergraduate Psychological Well-Being and Academic Motivation Dataset (MUPWAM).

A summary of constructs, corresponding instruments, and item composition is provided in Table 1, while the overall dataset structure and file organization are illustrated in Figure 1 to facilitate clarity and reuse.

**Table 1:** Summary of constructs, instruments, and item composition.

Construct	Instrument (Reference)	No. of items	Subdimensions	Scale range
Psychological WellBeing	Ryff (1989) Psychological WellBeing Scale	18	Autonomy, Environmental Mastery, Personal Growth, Positive Relations, Purpose in Life, Self-Acceptance	1–5
Academic Motivation	Vallerand et al. (1992) Academic Motivation Scale (AMS-C)	28	Intrinsic Motivation, Extrinsic Motivation, Amotivation	1–5
Coping Mechanisms	Carver (1997) Brief COPE	28^*^ (aggregated into 14 subscales)	Active Coping, Planning, Positive Reframing, Acceptance, Humor, Religion, Emotional Support, Instrumental Support, Self-Distraction, Denial, Venting, Substance Use, Behavioral Disengagement, Self-Blame	1–4/1–5^**^
Student-Life Stress	Gadzella (1994) Student-Life Stress Inventory (SLSI)	30	Academic Stressors, Personal Stressors, Environmental Stressors	1–5

^*^Brief COPE consists of 28 items grouped into 14 two-item subscales.

^**^Scale range harmonized to 1–5 in composite scoring for consistency across constructs.

All instruments were selected based on established validity and relevance to higher education and were adapted for clarity within the Malaysian undergraduate context.

The dataset consists of four primary constructs psychological wellbeing, academic motivation, coping mechanisms, and student-life stress each measured באמצעות validated multi-item instruments and organized into corresponding subscales. Demographic variables (e.g., gender, age, academic year, faculty cluster, and institution type) are included to support subgroup and comparative analyses. Raw item-level responses are processed into composite subscale and construct-level scores. The dataset is distributed across three main files: (1) a cleaned data file containing all variables and computed scores, (2) a codebook detailing variable definitions and coding schemes, and (3) a README file describing methodology and data usage. This structured organization supports reproducibility, secondary analysis, and integration with statistical software.

### 3.2 Variable composition

Each participant record contains 68 observed variables, categorized as follows:

- Demographics: gender, age, year of study, faculty cluster, and institution type
- Psychological WellBeing: 18 items across six subscales
- Academic Motivation: 28 items covering intrinsic motivation, extrinsic motivation, and amotivation
- Coping Mechanisms: 14 subscales derived from the Brief COPE
- Student-Life Stress: 30 items assessing academic, personal, and environmental stressors

Composite mean scores for each construct were calculated and included as standardized variables (range: 1–5).

Missing data were minimal (< 2%) and were addressed using mean substitution within subscales. This approach was adopted to preserve sample size and maintain consistency in descriptive and correlational analyses. However, mean substitution may attenuate variance and introduce bias, particularly in multivariate modeling. Accordingly, future users are encouraged to consider more robust approaches such as multiple imputation or expectation-maximization techniques when conducting advanced analyses.

### 3.3 Descriptive statistics

Descriptive analyses indicate generally moderate to high levels of psychological wellbeing (*M* = 3.87, *SD* = 0.61) and academic motivation (*M* = 3.74, *SD* = 0.68). Coping mechanisms were observed at moderate levels (*M* = 3.45, *SD* = 0.57), while student-life stress levels were comparatively higher (*M* = 3.12, *SD* = 0.64).

These statistics provide an overview of central tendencies and variability within the sample and are intended as descriptive reference points for subsequent research. Given the cross-sectional design, these findings should be interpreted as associational rather than causal, and no directional inferences should be made.

### 3.4 Data quality and reliability

All scales demonstrated satisfactory internal consistency:

- Psychological WellBeing (α = 0.89)
- Academic Motivation (α = 0.94)
- Coping Mechanisms (α = 0.93)
- Student-Life Stress (α = 0.82)

Composite reliability values exceeded 0.80, and Average Variance Extracted (AVE) values were generally above 0.50, indicating adequate convergent validity.

As all constructs were measured self-report instruments, the dataset may be subject to common method variance (CMV). This limitation should be considered when interpreting relationships among variables. For advanced analyses, researchers are encouraged to apply statistical remedies such as marker variable techniques or latent method factor approaches to assess and control for CMV effects.

### 3.5 Correlation structure

Zero-order correlations among the primary constructs were computed using Pearson's *r* to provide an overview of relationships within the dataset. Psychological wellbeing, coping mechanisms, and academic motivation were positively associated, whereas student-life stress showed negative associations with these variables.

Correlation coefficients ranged from *r* = 0.25 to *r* = 0.65, indicating small to moderate relationships consistent with theoretical expectations. These relationships are associational in nature and should not be interpreted as evidence of causality. The reported correlation matrix may serve as a reference for replication, comparative, or meta-analytic studies.

### 3.6 Reuse potential

The MUPWAM dataset offers a flexible resource for a wide range of research applications in educational psychology, mental health, and higher education studies. The structured variables and standardized measures enable:

- Cross-cultural and cross-national comparisons of student wellbeing and motivation
- Psychometric validation and scale adaptation in diverse populations
- Multivariate modeling, including regression, mediation, moderation, and structural equation modeling (SEM)
- Exploratory analyses of stress, coping, and academic engagement

Users should consider the following when reusing the dataset:

- Findings should be interpreted as associational rather than causal
- Sampling limitations may affect generalizability
- Common method variance (CMV) may influence observed relationships

For subgroup analyses, researchers are strongly encouraged to conduct measurement invariance testing (e.g., across gender or academic discipline) to ensure comparability of constructs.

## Data Availability

The datasets presented in this study can be found in online repositories. The names of the repository/repositories and accession number(s) can be found below: https://zenodo.org/records/16964826?utm_source=chatgpt.com.
